# Noncoding RNA Mediated Traffic of Foreign mRNA into Chloroplasts Reveals a Novel Signaling Mechanism in Plants

**DOI:** 10.1371/journal.pone.0012269

**Published:** 2010-08-19

**Authors:** Gustavo Gómez, Vicente Pallás

**Affiliations:** Instituto de Biología Molecular y Celular de Plantas (IBMCP), Consejo Superior Investigaciones Científicas (CSIC) - Universidad Politécnica de Valencia (UPV), Valencia, Spain; East Carolina University, United States of America

## Abstract

Communication between chloroplasts and the nucleus is one of the milestones of the evolution of plants on earth. Proteins encoded by ancestral chloroplast-endogenous genes were transferred to the nucleus during the endosymbiotic evolution and originated this communication, which is mainly dependent on specific transit-peptides. However, the identification of nuclear-encoded proteins targeted to the chloroplast lacking these canonical signals suggests the existence of an alternative cellular pathway tuning this metabolic crosstalk. Non-coding RNAS (NcRNAs) are increasingly recognized as regulators of gene expression as they play roles previously believed to correspond to proteins. Avsunviroidae family viroids are the only noncoding functional RNAs that have been reported to traffic inside the chloroplasts. Elucidating mechanisms used by these pathogens to enter this organelle will unearth novel transport pathways in plant cells. Here we show that a viroid-derived NcRNA acting as a 5′UTR-end mediates the functional import of Green Fluorescent Protein (GFP) mRNA into chloroplast. This claim is supported by the observation at confocal microscopy of a selective accumulation of GFP in the chloroplast of the leaves expressing the chimeric vd-5′UTR/GFP and by the detection of the GFP mRNA in chloroplasts isolated from cells expressing this construct. These results support the existence of an alternative signaling mechanism in plants between the host cell and chloroplasts, where an ncRNA functions as a key regulatory molecule to control the accumulation of nuclear-encoded proteins in this organelle. In addition, our findings provide a conceptual framework to develop new biotechnological tools in systems using plant chloroplast as bioreactors. Finally, viroids of the family Avsunviroidae have probably evolved to subvert this signaling mechanism to regulate their differential traffic into the chloroplast of infected cells.

## Introduction

Chloroplasts are the hallmark organelle of photosynthetic eukaryotes [1], [2]. They are responsible for many metabolic processes, including photosynthesis and biosynthesis of diverse essentials primary and secondary metabolites [3]–[5]. These organelles arose from cyanobacterium-like ancestors that were engulfed by eukaryotic cells during the endosymbiotic evolution [6]. Although chloroplasts have maintained remnants of the ancestral genome, the majority of the genes encoding chloroplast proteins have been transferred to the plant genome. Consequently, most of the proteins found in this organelle are nucleus-encoded [1], [2], [7].

The exchange of genetic information between the nucleus and the chloroplast has resulted in a close coordination in the activities of these two organelles during plant development which is mediated by both anterograde (nucleus-chloroplast) and retrograde (chloroplast-nucleus) signals [2], [5]. Thus, signaling between plastids and nucleus is required to maintain chloroplast biological functions. It is widely accepted that this genome-coordinated mechanism is mediated by proteins encoded in the nucleus, synthesized in the cytosol in the corresponding precursor form (containing a targeting signal called transit peptide, TP) and then imported into the organelle [7]. However, the identification, in recent years of a significant number of nuclear-encoded proteins lacking canonical transit-peptides [8], [9] supports the existence of alternative pathways in plant cell that tune the accumulation of host proteins in chloroplasts [1].

High-throughput sequencing methods have unveiled a complex network of transcripts derived from the eukaryotic genome that include a large number of noncoding RNAs (ncRNAs) [10]–[12]. NcRNAs are increasingly recognized as regulators of gene expression and the other cellular functions, playing roles previously believed to correspond to proteins [10]–[12]. Although ncRNAs were thought to be restricted to the nuclear or cytosolic compartments of the cell, these regulatory RNAs were also identified in mitochondria and chloroplasts mapping to intergenic regions of the organellar genome [13].

Viroids are small ncRNAs that infect plants [14]–[16]. They are classified into two families: *Pospiviroidae* and the *Avsunviroidae*. Members of the family *Avsunviroidae* replicate and accumulate in the chloroplast and are the only functional RNAs that have been reported to traffic inside this organelle [15], [16]. Elucidating mechanisms used by these pathogens to enter chloroplasts will unearth novel transport pathways in plant cells.

Here we report that a viroid-derived ncRNA sequence acting as 5′UTR (vd-5′UTR) mediates the specific import of a functional GFP mRNA into the chloroplasts of *N. benthamiana* cells, thus supporting the existence of a novel signaling mechanism between the host cell and these organelles.

## Results and Discussion

**Figure 1:**
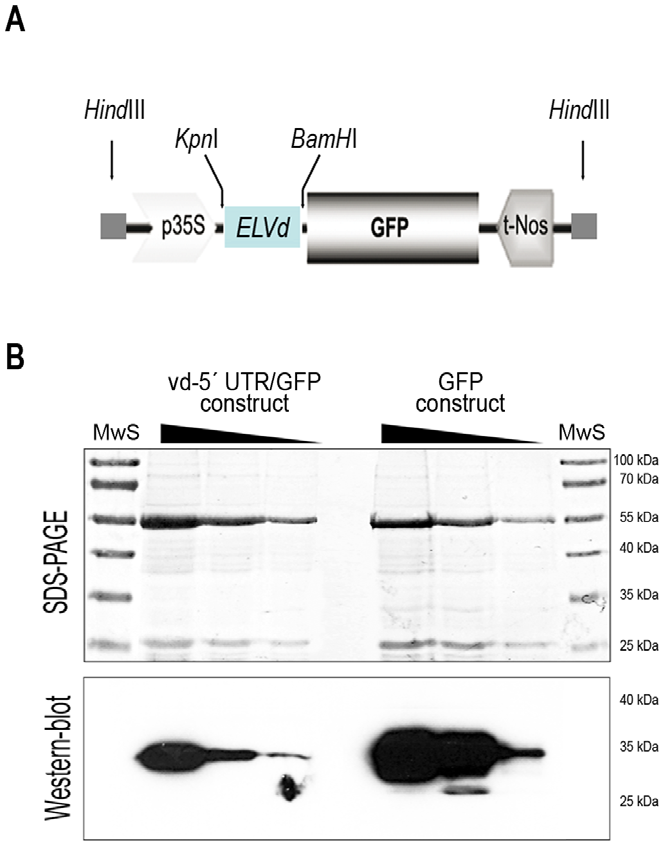
Transcriptional fusion of viroid derived 5′UTR end to GFP cDNA. (**A**) Physical map of the vd-5′UTR/GFP construct. (**B**) Serological detection of the vd-5′UTR/GFP. Total proteins were extracted from infiltrated leaves, electrophoresed in 10% SDS-PAGE (upper panel) and blotted for serological detection (lower panel). The vd-5′UTR/GFP and the GFP were clearly detected and show similar relative electrophoretic mobility, indicating that the viroid derived sequence acts as a true untranslated RNA. Three 1∶5 serial dilutions are sown for each construct. MwS: Molecular weight standard.

**Figure 2:**
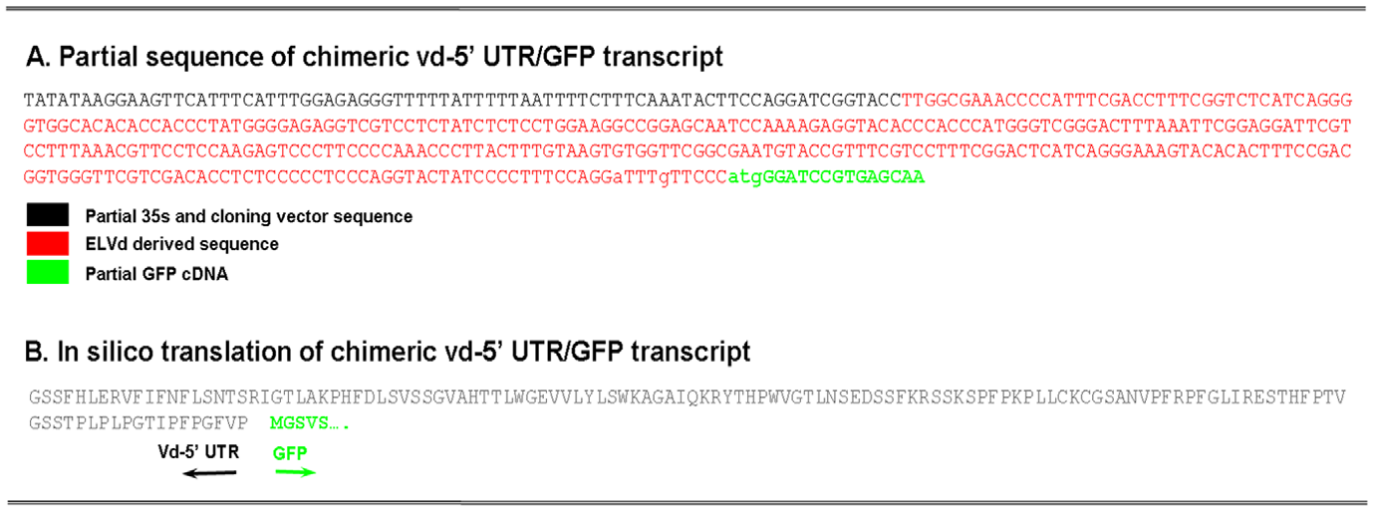
Details of the fusion ELVd-derived RNA/GFP mRNA. (**A**) Partial DNA sequence of the vd-5′UTR-GFP construct (AN - HM136583) used in this study. (**B**) In silico predicted translation of the vd-5′UTR-GFP transcript.

The use of transcriptional fusion between viroid derived sequences and the mRNA of a reporter protein were previously described to analyze plant-specific cellular processes such as RNA silencing or viroid-induced pathogenesis [17], [18]. Consequently, to evaluate if an ncRNA could regulate the transport of mRNAs to chloroplasts, we constructed a chimeric DNA containing a modified *Eggplant latent viroid* (ELVd) [19] cDNA sequence fused as an untranslated region (UTR) to the 5′end of the cDNA of the Green Fluorescence Protein (GFP) (Figure 1A). The fused viroid-GFP cDNA (vd-5′UTR-GFP) was cloned in a binary vector and transfected into *Agrobacterium tumefaciens*. The correct transcriptional fusion of the vd-5′UTR-GFP fragments was checked by DNA sequencing and by *in silico* translation of the chimeric mRNA (Figure 2). The functionality of this chimeric transcript was analyzed by comparing its transient expression with that of the unmodified GFP mRNA in *N. benthamiana* plants in agroinfiltration assays. Total proteins were extracted from agroinfiltrated leaves and analyzed by Western blot assays. As observed in Figure 1B the unmodified GFP and vd-5′UTR-GFP, show a similar relative electrophoretic mobility, indicating that the viroid-derived ncRNA act in this chimeric transcript as a true 5′ untranslated region of the GFP mRNA, thus verifying the prediction performed *in silico*.

**Figure 3:**
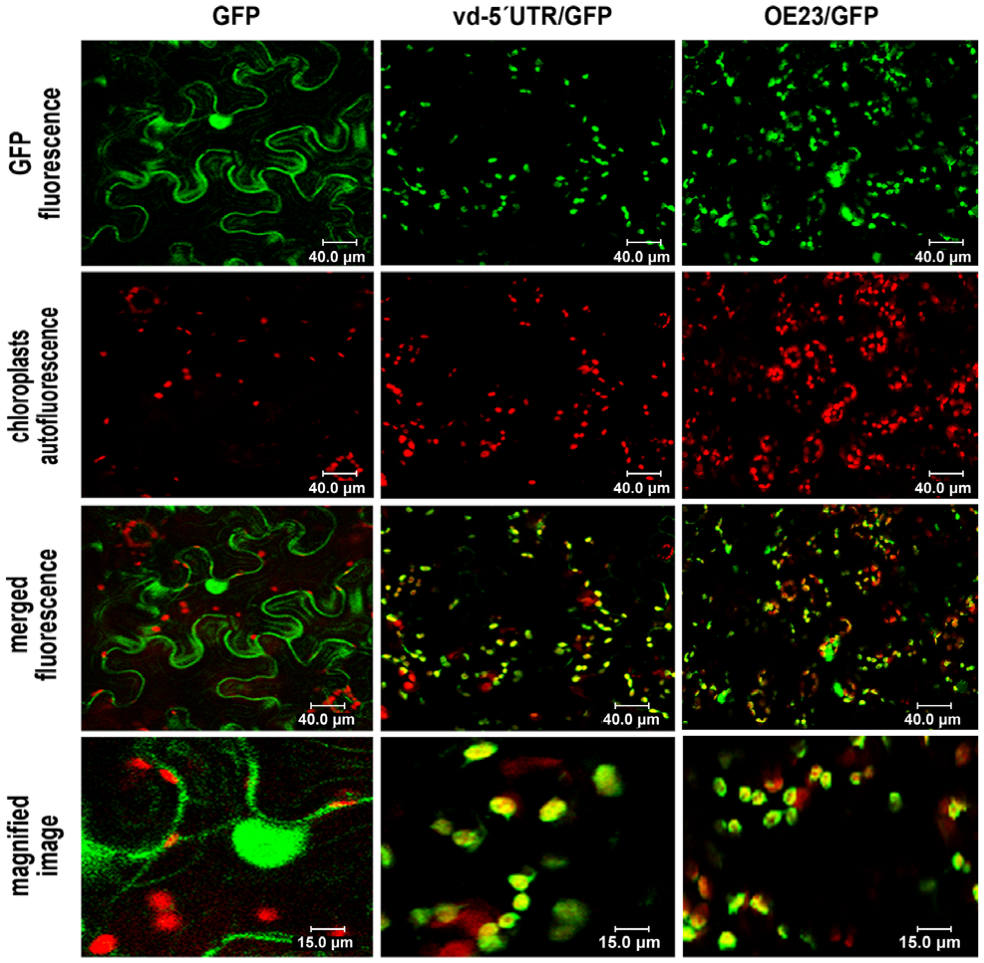
The GFP arising from vd-5′UTR/GFP transcripts accumulate specifically in chloroplasts. Confocal microscope observation of the *N. benthamiana* leaves expressing unmodified GFP (left panels), vd-5′UTR-GFP (central panels) or OE23/GFP (right panels). As observed, the vd-5′UTR/GFP mimics the cellular localization of the OE23/GFP construct that accumulates specifically in chloroplasts.

When the infiltrated plants were analyzed by confocal microscopy we observed that the unmodified GFP (used as a control) was uniformly distributed in the nucleus and cytoplasm of the examined cells (Figure 3, left panels), while the GFP arising from the vd-5′UTR/GFP transcript was largely localized in the chloroplasts (Figure 3, central panels). The chloroplastic localization of the vd-5′UTR/GFP was confirmed by a comparative analysis using a GFP fused to a chloroplast-specific transit peptide derived of a protein of the oxygen evolving complex (OE23) as reference [20]. As observed in Figure 3 the vd-5′UTR/GFP mimics the cellular localization of the OE23/GFP construct that specifically accumulated in the chloroplasts of *N. benthamiana* cells (right panels) thus confirming that the GFP translated from the chimeric transcript vd-5′UTR/GFP accumulates specifically in this organelle.

**Figure 4:**
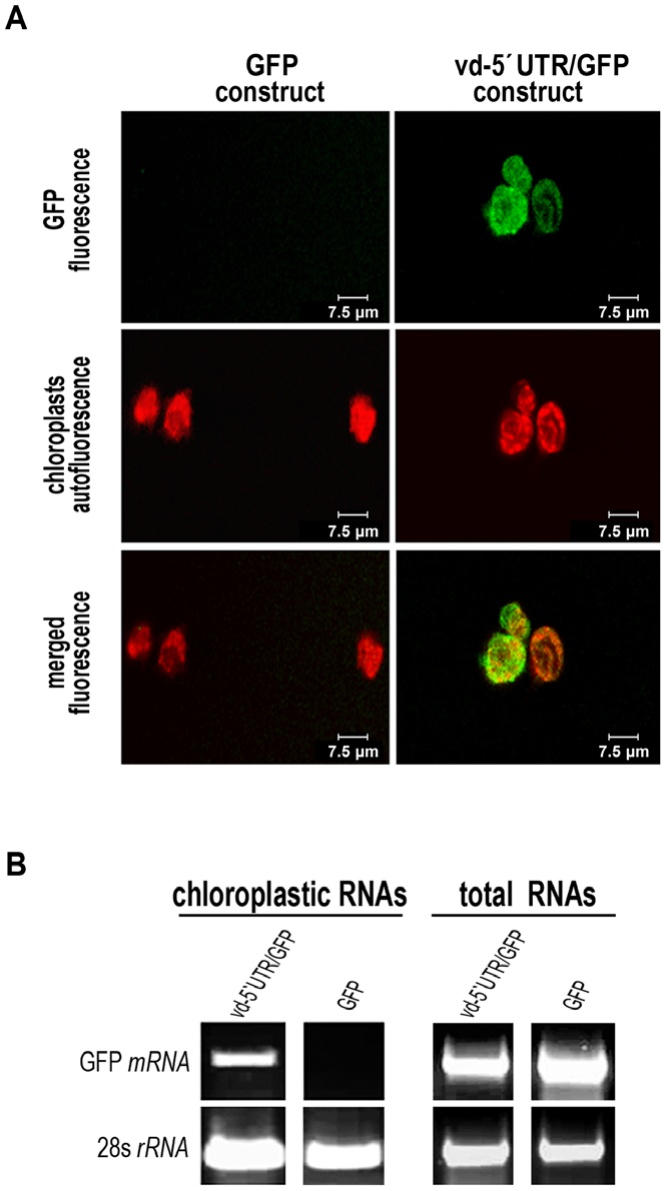
Vd-5′UTR mediates the traffic of functional GFP-mRNA to chloroplasts. (**A**) Confocal microscope observation of chloroplasts isolated from leaves expressing GFP or vd-5′UTR/GFP. (**B**) RT-PCR analysis of GFP transcripts extracted from chloroplasts isolated from leaves infiltrated with both constructs. Chloroplast ribosomal RNA 28 s was amplified as a control of isolated-chloroplasts integrity.

To demonstrate the chloroplastic localization of the chimeric transcripts, we isolated chloroplasts from *N. benthamiana* cells expressing the GFP or vd-5′UTR/GFP constructs (Figure 4A), and analyzed the accumulation of GFP mRNA in these organelles by RT-PCR. As seen in Figure 4B (upper panel), the GFP transcripts were detected in the chloroplasts isolated from the cells expressing the vd-5′UTR/GFP, but not in those isolated from the cells expressing unmodified GFP. The 28 s rRNA used as a control of isolated-chloroplasts integrity was similarly detected in both GFP mRNA-accumulating and non GFP mRNA-accumulating organelles (Figure 4B, bottom panel). This finding provides direct evidence that the vd-5′UTR-GFP mRNA transcribed in the nucleus of *N. benthamiana* cells is specifically targeted to chloroplasts where, according to the confocal microscopy experiments, it is efficiently retained and translated in functional GFP.

In short, our results demonstrate that an ncRNA fused as a 5′UTR end acts as a key regulatory molecule which is able to mediate the specific trafficking of a functional foreign mRNA into the chloroplast, thus supporting the existence of a novel signaling mechanism between the host cell and chloroplasts. This emergent paradigm highlights a novel host-modulated regulatory mechanism that would be potentially able to control the gene expression and the accumulation of the nuclear-encoded proteins in chloroplasts, as an alternative to that based on transit-peptides. Interestingly, the search for entirely new mechanisms involved in this crucial transport system has been recently enunciated as a major priority for future research in the field of chloroplasts protein targeting [2]. In this context, it is reasonable to assume that the *Avsunviroidae* family viroid members have probably evolved to subvert this signaling mechanism in order to regulate their differential traffic and accumulation into the chloroplast of infected plant cells.

Indirect evidence may suggest that, at least in parasitic plants which plastid genome lacks most, if not all, of the photosynthetic genes, tRNAs must be imported from outside the plastid [21]. In a previous work it was shown that the mRNA coding for the eukaryotic translation factor 4E, an essential regulator of translation, enters the chloroplasts in four different plant species [22]. In addition, the localization in the chloroplasts of an heterologous GFP mRNA fused to the eIF4E RNA was also observed. However, the eIF4E protein was not detected in the chloroplast and thus, to date, the import of functional RNAs into chloroplasts has not been directly demonstrated. Our results demonstrate for the first time that an RNA sequence is able to transport to chloroplasts *per se* and that it is functional in this organelle.

Finally, the use of plant chloroplasts as bioreactors has emerged in the last years as a valuable biotechnological alternative to microbial fermentation and mammalian cell culture for the industrial production of diverse compounds [23]–[26]. Consequently, the description of a ncRNA acting as an untranslated signal capable of mediating the stable expression of foreign proteins in the chloroplast provides a conceptual framework to develop alternative strategies in the production of biopharmaceuticals or vaccines in systems using plant chloroplasts as bioreactors.

## Materials and Methods

### Plasmid construction

The viroid-derived cDNA fragment was obtained by PCR, starting from the cDNA of ELVd (AN - AJ536613) [19]. The amplified ELVd fragment was cloned in a binary plasmid pMOG 800 carrying the GFP cDNA under the control of the *Cauliflower mosaic virus* 35S promoter and the nopaline synthase terminator (t-Nos) [27]. The resultant vector vd-5′UTR/GFP (AN - HM136583) contains an ELVd derived cDNA fused as untranslated region (UTR) to the 5′end of the GFP cDNA. The expression of the fusion vd-5′UTR/GFP was checked by Western blot assays as previously described [17].

### Western blot assays

Total proteins were extracted by grinding four leaf discs (5.5 mm diameter) in 100 µl of protein extraction buffer (150 mM Tris, pH 8.8, 5% SDS, 15% glycerol, 100 mM DTT). The homogenate was centrifuged for 1 min at 12.000 rpm in a micro centrifuge and the supernatant collected. The supernatant (30 µl) was denatured and fractionated by 10% SDS-PAGE and transferred to Hybond™-P membranes (GE Healthcare Life Sciences). Membranes were blocked for 1 h [TBS (500 mM NaCl, 20 mM Tris, pH 7.5), 5% defatted milk, 2% BSA, 0.3% Tween 20] and incubated overnight at 4°C with an Anti-Green fluorescent protein (GFP) mouse IgG monoclonal antibody (ROCHE Diagnostics GmbH, Manneiheim, Germany). Membranes were washed (TBS, 0.3% Tween 20), incubated with ECL™ Peroxidase labeled anti-mouse antibody, and revealed by luminescence (ECL+Plus, GE Healthcare Life Sciences) according to the manufacturer instructions. A similar construct carrying unmodified GFP was used as control.

### Agroinfiltration

The *N. benthamiana* plants were infiltrated with the 5′UTR-GFP or GFP constructs as previously described [17] and maintained in environmentally controlled growing chambers (28°C, 14 h of light). The GFP expression in plants was analyzed at 72 and 96 hours after agroinfiltration with a TCS SL confocal laser scanning microscope (Leica), with excitation at 488 nm and emission at 510–560 nm. To confirm the chloroplastic localization of the vd-5′UTR/GFP, we performed a comparative analysis using as reference a GFP fused to chloroplast-specific transit peptide derived of a protein of the Oxygen evolving complex (OE23) [20].

### Chloroplast isolation

Chloroplasts were isolated from plants infiltrated with the vd-5′UTR/GFP or GFP constructs respectively, following a modification of a previous protocol [28]. Leaves (10 g) were homogenized in Extraction buffer (300 mM Sorbitol, 1 mM MgCl2, 50 mM HEPES/KOH [pH 7.8], 2 mM EDTA, 004% β-mercaptoethanol and 0.1% polyvinilpyrrolidone). The homogenate was filtered through miracloth layers and centrifuged 10 min. at 1500 g. The pellets were resuspended in 4 ml of Isolation buffer (300 mM Sorbitol, 1 mM MgCl2, 50 mM HEPES/KOH [pH 7.8] and 2 mM EDTA), loaded in a Percoll step gradient (15% – 35% and 55%) and centrifuged 20 min. at 8000 g. Intact chloroplast were recovered from the 35%–55% interphase, washed with isolation buffer and collected by centrifugation (5 min. at 1000 g). The pellet containing the chloroplast was used to extracts chloroplastic RNAs.

### RNA isolation and RT-PCR analysis

The RNAs were extracted from isolated chloroplasts and *N. benthamiana* leaves using TRI reagent (SIGMA, St. Louis, MO, USA) according to the manufacturer instructions. RT-PCR analysis was performed as previously described [16] using specific primers to amplify GFP mRNA and a region of 28 s chloroplast ribosomal RNA.
